# Human Platelet Lysate Supports Efficient Expansion and Stability of Wharton’s Jelly Mesenchymal Stromal Cells via Active Uptake and Release of Soluble Regenerative Factors

**DOI:** 10.3390/ijms21176284

**Published:** 2020-08-31

**Authors:** Mariana Cañas-Arboleda, Karl Beltrán, Carlos Medina, Bernardo Camacho, Gustavo Salguero

**Affiliations:** Advanced Therapies Unit, Instituto Distrital de Ciencia Biotecnología e Innovación en Salud—IDCBIS, 111611 Bogotá, Colombia; mcanas@idcbis.org.co (M.C.-A.); kbeltran@idcbis.org.co (K.B.); cmedina@idcbis.org.co (C.M.); bacamacho@idcbis.org.co (B.C.)

**Keywords:** Wharton’s Jelly Mesenchymal Stromal Cells, human platelet lysate, cytokines, human growth factors

## Abstract

Manufacturing of mesenchymal stromal cell (MSC)-based therapies for regenerative medicine requires the use of suitable supply of growth factors that enhance proliferation, cell stability and potency during cell expansion. Human blood derivatives such as human platelet lysate (hPL) have emerged as a feasible alternative for cell growth supplement. Nevertheless, composition and functional characterization of hPL in the context of cell manufacturing is still under investigation, particularly regarding the content and function of pro-survival and pro-regenerative factors. We performed comparative analyses of hPL, human serum (hS) and fetal bovine serum (FBS) stability and potency to support Wharton’s jelly (WJ) MSC production. We demonstrated that hPL displayed low inter-batch variation and unique secretome profile that was not present in hS and FBS. Importantly, hPL-derived factors including PDGF family, EGF, TGF-alpha, angiogenin and RANTES were actively taken up by WJ-MSC to support efficient expansion. Moreover, hPL but not hS or FBS induced secretion of osteoprotegerin, HGF, IL-6 and GRO-alpha by WJ-MSC during the expansion phase. Thus, hPL is a suitable source of factors supporting viability, stability and potency of WJ-MSC and therefore constitutes an essential raw material that in combination with WJ-MSC introduces a great opportunity for the generation of potent regenerative medicine products.

## 1. Introduction

Application of cell therapies based on the use of mesenchymal stromal cells (MSC) has gained a major place among current approaches for regenerative medicine worldwide. Advances in the adoption of new technologies and procedures oriented to the manufacturing of MSC-based medicinal products have been pivotal to speed-up translation into clinical settings of a wide variety of therapeutic tools from tissue engineering to immunotherapy. In this context, optimizing cell manufacturing workflow is a critical step to obtain better and more effective therapies by ensuring high yield, viability, reproducibility and safety of the active cellular product. Efficiency of cell manufacturing process depends on the use of suitable cell supplements to support MSC expansion [1]. Fetal bovine serum (FBS) has been for decades the source of growth factors for a wide range of cell culture applications including cell therapies. However, the presence of xenogeneic antigens in FBS preparations rises several concerns regarding unwanted effects linked to pre-immunization priming, which ultimately will impact on the therapeutic efficacy of the final cell product [2]. Additional issues regarding pathogen safety, animal welfare, costs and complex supply chain associated to FBS production, have enforced the search of additional alternatives for growth supplements applied to cell manufacturing [2].

Blood derivatives as alternative for FBS have gained attention over the last decade. Among them, human blood components such as plasma, serum and platelet concentrates (frozen/thawed lysates or serum-converted) are currently being investigated for MSC-based cell therapy production [1,3]. Plasma and platelet-poor plasma obtained from anticoagulated total blood was initially employed as supplement for ex-vivo expansion of MSC and other types. Later, human serum was used as medium additive for human cell growth by taking advantage of its higher platelet content and endogenous thrombin activity, leading to increased concentrations of platelet-derived soluble factors [1,3]. Remarkably, the use of platelet concentrates has currently expanded in the field of MSC production, as they have shown superior potency for cell expansion while allowing large scale, cost-effective and standardized production of supplement batches for cell manufacturing. Among them, human platelet lysate (hPL) constitutes one of the most powerful xeno-free supplements employed in the culture of MSC, demonstrated by its proven capacity to promote cell growth and improve cell survival, proliferation and preservation of the MSC phenotype [4]. In fact, hPL-based supplements supported colony-forming unit formation, enhanced osteoblastic differentiation potential, maintained genetic stability and prompted potent immunomodulatory effects of diverse MSC types [5,6,7]. Finally, MSC cell manufacturing in the presence of hPL has been readily used in clinical scale production in several trials using MSC-based therapies [8]. Noteworthily, hPL has been recognized for its high content of growth factors that trigger in angiogenic processes, cell repair and macrophage activation processes required for tissue regeneration [9,10,11]. From the manufacturing point of view, hPL can be obtained from buffy coat, platelet-rich plasma or apheresis concentrates. Importantly, the use of allogeneic donors has become an attractive approach for hPL generation, exhibiting several advantages over autologous preparations such as low batch-to-batch variations via donor pooling, increased volume availability, reproducibility during production and affordable manufacturing and release process [4,8]. However, larger pool sizes might also enhance the risk of transmitting infectious agents, introducing the need of inactivation or removal of viruses during hPL production [8,12,13]. More importantly, correct characterization of pooled-hPL batches in terms of potency, stability and content, has not been fully explored in the context of MSC production.

Wharton’s jelly (WJ) MSC are being intensively explored as “off-the-shelf” cell therapy for a wide array of chronic degenerative disorders including autoimmune and orthopedic conditions. In this context, generation of fully characterized, already-available WJ-MSC allogeneic cell banks has broadened the application potential of MSC products, thereby accelerating their clinical translation for regenerative medicine [14,15]. Thus, optimizing WJ-MSC production process by introducing highly reproducible and sufficient growth factor supply is critical to reach sustained and effective cell batch production for further clinical use. Importantly, hPL pools have shown superior performance of WJ-MSC proliferation as compared to FBS [16]. Moreover, no differences between blood groups and soluble factors content were detected in several tested hPL batches [17]. Even though incremental evidence indicates the feasibility of using hPL as a supplement for WJ-MSC-based production, little is known about the content of specific growth factors critical for WJ-MSC expansion. More importantly, the kinetics of uptake and secretion of essential growth factors by WJ-MSC under hPL-stimulation have not been thoroughly assessed. In this study, we conducted a comprehensive characterization of hPL batches regarding growth factor content and stability and compared them with human serum hS and FBS growth supplements. We hypothesized that hPL provides critical factors that are taken up by WJ-MSC to enhance their regenerative potential via constitutive secretion of pro-survival and immunomodulatory signals.

## 2. Results

### 2.1. Production of hPL and hS for Culture of WJ-MSC

Setting up an optimized workflow for the generation of reproducible starting material required for cell production, is critical to ensure consistent and reliable growth rates of WJ-MSC for clinical scaling. Here we first evaluated leukocyte-poor platelet (LPP) pools obtained from O+ LPP bags collected by the institutional blood bank. LPP from three donors per batch were initially pooled and platelets, leukocytes, erythrocytes and pH were recorded. In average, we observed 10 × 10^5^ platelets/µL present in evaluated LPP pools (*n* = 16) and as expected, we detected erythrocyte and leukocyte levels below 8 × 10^2^/µL and 6 × 10^2^/µL respectively (Table 1).

**Figure 1:**
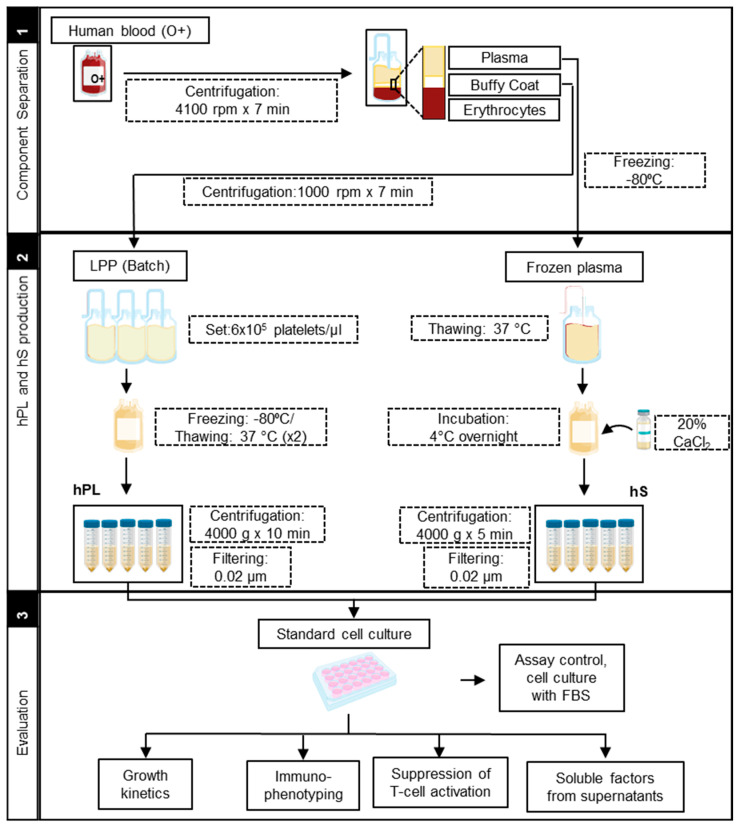
Manufacturing protocol scheme of human platelet lysate (hPL) and human serum (hS).

To get homogenous LPP pools, we adjusted the platelet concentration to 6 × 10^5^/µL. By doing so, we were able to reduce the variability of platelet content from 25% in original, up to 3% in adjusted LPP pools. Importantly, adjusted pools also showed reduction of leukocytes (35%) and erythrocytes (48.4%) levels. LPP pools displayed neutral pH (average of 7 ± 0.5) with a low coefficient of variability (6%) between batches. In summary, we were able to generate LPP pools with standard concentrations of platelets and low inter-batch variability, thereby favoring the manufacture of homogenous hPLs. Accordingly, LPP pools showed lower levels of erythrocytes and leukocytes and neutral pH, providing low immunogenicity and metabolic balance ideal for proliferation of WJ-MSC. After LPP characterization, we further produced hPL batches following a workflow established in house (Figure 1).

**Figure 2:**
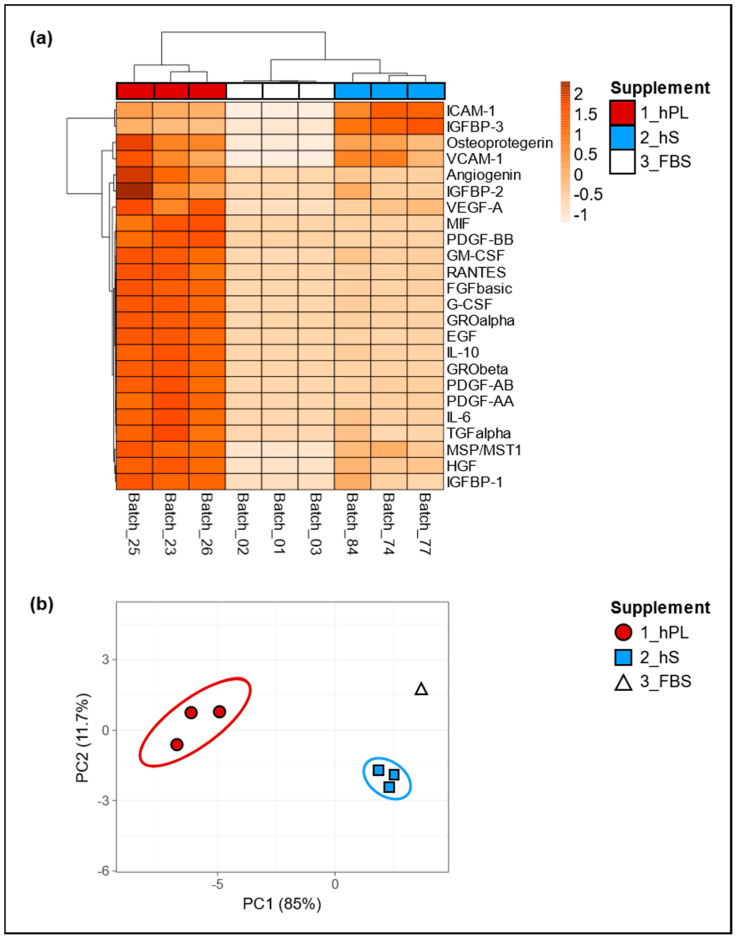
Secretome characterization of hPL, hS and fetal bovine serum (FBS)**.** Global differences in cytokine concentrations in supernatants from hPL, hS and FBS were compared and visualized by (**a**) unsupervised hierarchical clustering, where rows correspond to factors clustered using correlation distances and average linkage. Columns depict clusters using Euclidean distance and average linkage; (**b**) two-dimensional Principal Component Analyses (PCA) was carried out to determine associations between expression patterns of analyzed factors. hPL *n* = 3, hS *n* = 3 and FBS *n* = 3.

Since we assumed that the biological effect of hPL as cell culture supplement depends on soluble factors including growth factors and cytokines, we determined the levels of selected factors present in three batches of hPL and compared them with hS (*n* = 3) and FBS (*n* = 3) batches, in order to evaluated concentration variability in all supplement types. Importantly, we observed pronounced increment in the levels of angiogenin, IGFBP-1 and 2, VEGF-A, MIF, PDGF-AA, AB and BB, GM- CSF, RANTES, FGF basic, G-CSF, GRO alpha and Beta, EGF, IL-10, IL-6, TGF alpha, MSP/MST1 and HGF in hPL as compared to hS and FBS (Figure 2a). Of note, hS and FBS batches showed similarly low levels in most of the evaluated factors except for ICAM-1, IGFBP-3, osteoprotegerin and VCAM-1 in were we observed significant enrichment in hS batches. Here, we observed an average concentration of ICAM-1 with an increase of 45.3% and 99.8% as compared to hPL and FBS respectively and in IGFBP-3 with an increase of 63.5% and 99.7% compared to hPL and FBS. For Osteoprotegerin and VCAM-1, we found hPL to show higher concentrations with an increase of 46.2% and 14.3% compared to hS and an increase of 99.4% and 99.8% compared to FBS, respectively for each of these factors (Table S1). By means of principal component analyses we confirmed that the first and second main components explained 11.7% and 85% of the associations for the analyzed factors present in hPL, hS and FBS, where three different clusters were clearly representing “hPL” “hS” and “FBS” groups (Figure 2b). Thus, the obtained clusters of soluble factors strongly indicate a unique molecular composition of hPL that was not present neither in hS nor in FBS. Importantly, we confirmed low variation in the concentration of evaluated factors among hPL batches resulting from homogeneous preparations derived from our approach.

### 2.2. Cytokine and Growth Factor Stability Present in hPL

**Figure 3:**
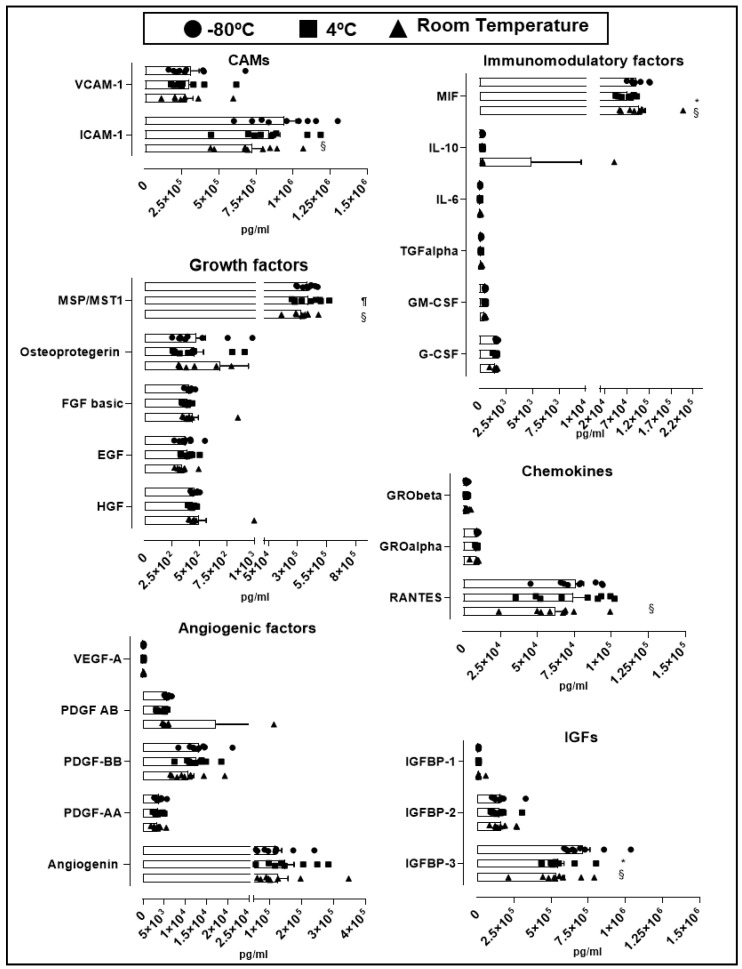
Stability of soluble factors at different storage conditions. Soluble factors were grouped by cellular adhesion molecule (CAMs), angiogenic factors, growth factors, insulin-like growth factors (IGFs), immunomodulatory factors and chemokines. Three storage conditions at −80 °C (circle, *n* = 9), 4 °C (square, *n* = 9) and room temperature (triangle, *n* = 9) were evaluated. Data is represented as mean ± SEM. § *p* < 0.05 −80 °C vs. room temperature, ¶ *p* < 0.05 4 °C vs. room temperature, * *p* < 0.05 −80 °C vs. 4 °C, analyzed by Tukey’s test.

An essential quality standard required for the use of hPL as culture supplement for expansion of GMP-grade WJ-MSC is linked to its stability under different storage conditions. We therefore assessed hPL stability according to the variation of soluble factors levels present in different batches stored at −80 °C for 30 days, to 4 °C for 30 days and at room temperature for 5 days. Looking at the overall variation of cytokines and growth factors contained in hPL baches (*n* = 9), we observed no significant change in the majority of factors in hPL kept at −80 °C, 4 °C and room temperature (Figure 3). We only observed differences in cell adhesion molecules (CAM) ICAM-1, where we found significant reduction in hPL stored at room temperature (7.3 × 10^5^ pg/mL) in comparison with −80 °C-stored hPL (9.4 × 10^5^ pg/mL, *p* = 0.041). Similarly, insulin-like growth factors (IGF) IGFBP-3 showed statistically significant reduction at room temperature (5.3 × 10^5^ pg/mL, *p* = 0.0007) and 4 °C-stored hPL (4.4 × 10^5^ pg/mL, *p* = 0.0003) as compared to hPL stored at −80 °C (7.1 × 10^5^ pg/mL). Of note, IGFBP-3 was the most abundant factor as compared to IGFBP-1 and 2. We also detected loss of the immunomodulatory factor MIF in hPL stored at 4 °C (7.3 × 10^4^ pg/mL) as compared to levels obtained in hPL stored at −80 °C (9.3 × 10^4^ pg/mL, *p* = 0.0004) and room temperature (9.8 × 10^4^ pg/mL, *p* < 0.0001). Finally, the cytokine RANTES present in hPL kept under room temperature showed reduced levels in comparison with frozen hPL (6.3 × 10^4^ pg/mL vs. 7.6 × 10^4^ pg/mL, *p* = 0.04). Taken together, this data demonstrated the preservation of most of the hPL-contained soluble factors at different storage conditions including room temperature, associated with very low inter-batch variation. Only few evaluated factors such as ICAM-1, IGFBP-1, MIF and RANTES were affected at room temperature, as compared to frozen hPL.

### 2.3. Comparative Assessment of hPL, hS and FBS in the Expansion of WJ-MSC

**Figure 4:**
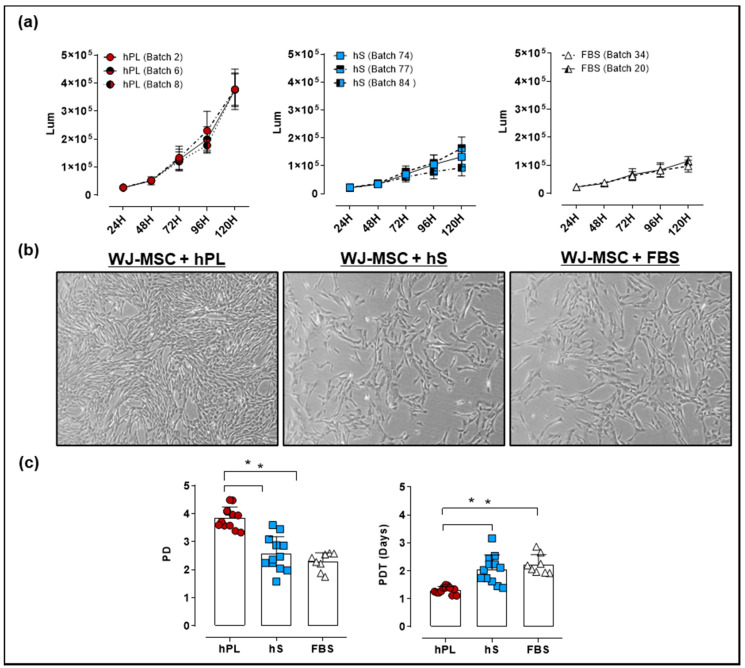
Evaluation of Wharton’s jelly (WJ)-mesenchymal stromal cell (MSC) growth expanded with 10% hPL, hS and FBS. (**a**) Proliferation Kinetics and viability of WJ-MSC expanded with different batches of hPL, hS and FBS determined by CellTiter-Glo Luminescent Cell Viability Assay (measurement units in Lum); (**b**) Representative micrographies of WJ-MSC showing cells in passage 2 expanded in hPL, hS and FBS after 4 days of culture (magnification 50X); (**c**) Population doubling (PD) and population doubling time (PDT) of WJ-MSC seeded with 10% hPL (green, *n* = 4 batches), hS (blue, *n* = 3 batches) and FBS (red, *n* = 2 batches). Data is represented as mean ± SD, * *p* < 0.05, analyzed by Tukey’s test.

For culture of WJ-MSC, an optimal supplement that potentiates cell proliferation in vitro is required. We isolated WJ-MSC from umbilical cords (UC) procured by the public umbilical cord blood bank program. For the generation of a master cell bank (MCB), WJ-MSC were isolated from 16 UC donors. We next evaluated the growth kinetics and viability of WJ-MSC expanded in culture media supplemented with hPL, hS and FBS by a luminescence-based Cell Viability Assay. After 24 and 48 h of culture there were no differences between WJ-MSC proliferation in the presence of hPL, hS or FBS. However, after 72 h of culture, we observed significant increase in the luminescence of WJ-MSC grown in hPL, as compared to hS (2.3 ± 0.65 fold-change, *p* < 0.0001) and FBS (2.7 ± 1.3 fold-change, *p* < 0.0001, Figure 4a).

Detected differences between media supplements were also evidenced in WJ-MSC confluency at 96 h, showing over 90% in presence of hPL as compared with hS (60% confluency) and FBS (60% confluency). Interestingly, cell morphology assessed in WJ-MSC showed a typical spindle-shape morphology with small cytoplasm in cells expanded in hPL, whereas cultured cells in hS and FBS displayed fibroblast-like shape and enlarged cytoplasm (Figure 4b). Accordingly, PD and PDT in hPL-cultured WJ-MSC were significantly higher as compared to hS (DP = 3.9 ± 0.4 and TDP = 1.3 ± 0.2 days vs. PD = 2.6 ± 0.6 and PDT = 2.1 ± 0.3 days respectively, *p* < 0.0001) (Figure 4c). Similarly, PD and PDT of WJ-MSC grown in hPL were significantly superior to cells expanded in FBS (PD = 2.3 ± 0.3 and PDT = 2.2 ± 0.1 days, *p* < 0.0001). Of note, WJ-MSC probed to hold genetic stability as shown by normal G-band pattern and absence of numeric or structural chromosomal alterations (Table S2). These data strongly support a superior capacity of hPL to promote WJ-MSC expansion while maintaining MSC identity in terms of cell surface markers and mesenchymal lineage differentiation.

### 2.4. Impact of hPL, hS and FBS in Cell Identity of WJ-MSC

**Figure 5:**
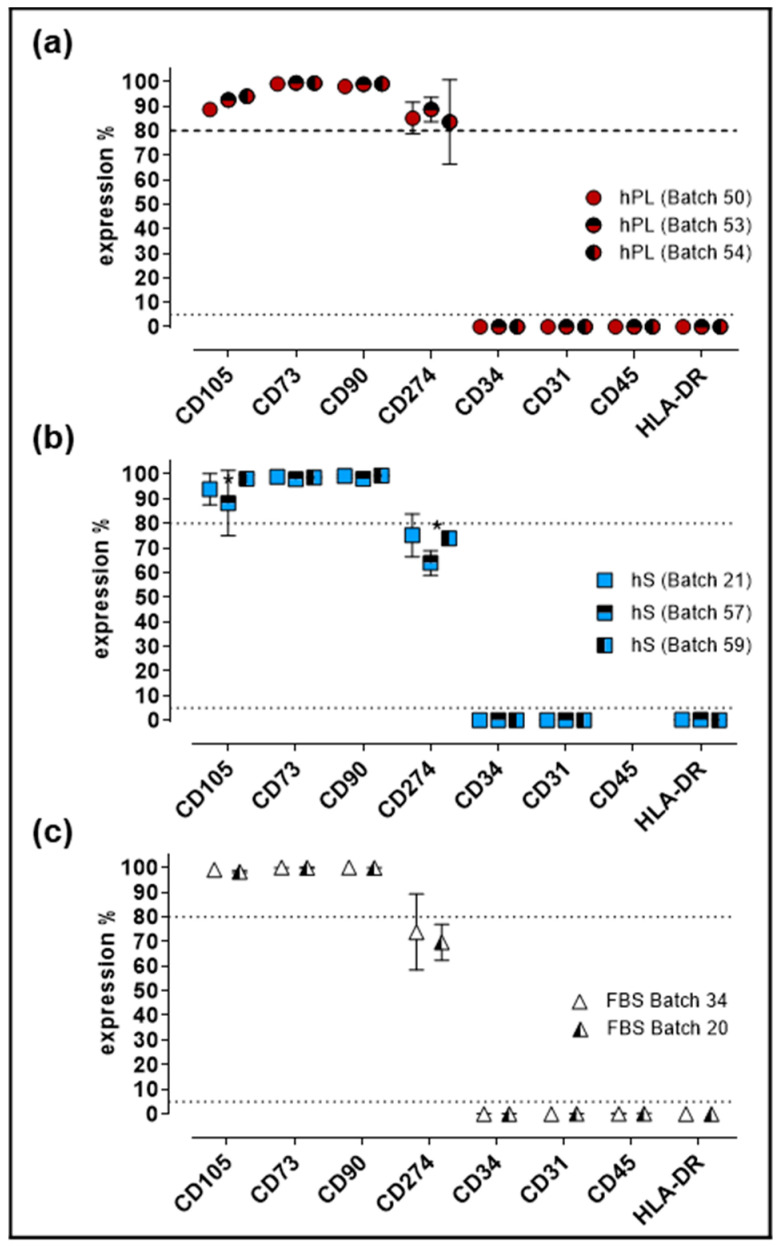
Immunophenotypic profile of WJ-MSC expanded with hPL, hS and FBS. (**a**) Percentage of expression of MSC markers present in WJ-MSC expanded with different batches of hPL (*n* = 3 batches); (**b**) hS (*n* = 3 batches) and (**c**) FBS (*n* = 2 batches); Data is shown as mean ± SD, * *p* < 0.05 analyze by Tukey’s test.

Evaluation of cell identity and purity is a critical parameter for GMP production of WJ-MSC. For quality control of established Master Cell Bank (MCB), we performed immunophenotypic characterization and evaluation of differentiation potential in cells expanded under hPL, hS and FBS supplement. Flow cytometry analysis showed that >80% of cells expressed CD105, CD73 and CD90 in WJ-MSC from all three groups (Figure 5).

Of note, significant increase of CD274-positive cell frequency was observed in hPL-stimulated WJ-MSC as compared to FBS and hS (*p* < 0.0001). Conversely, CD34, CD31, CD45 and HLA-DR expression was detected in less than 2% of the cells in all evaluated groups. Finally, cells expanded in hS presented significant reduction of CD105-positive cells (batch 57 and 59, *p* = 0.0055) and CD274-positive WJ-MSC in batch 57 versus 21 (*p* = 0.0015) and 59 (*p* = 0.0051, Figure 5b). Consistent with previous observations, WJ-MSC from a large MCB produced with hPL as supplement, displayed stable immunophenotype profile (Figure S1) and were able to differentiate into osteogenic, adipogenic and chondrogenic lineage (Figure S2).

### 2.5. WJ-MSC Expanded with hPL, hS and FBS Maintain their Immune Suppressive Potency

**Figure 6:**
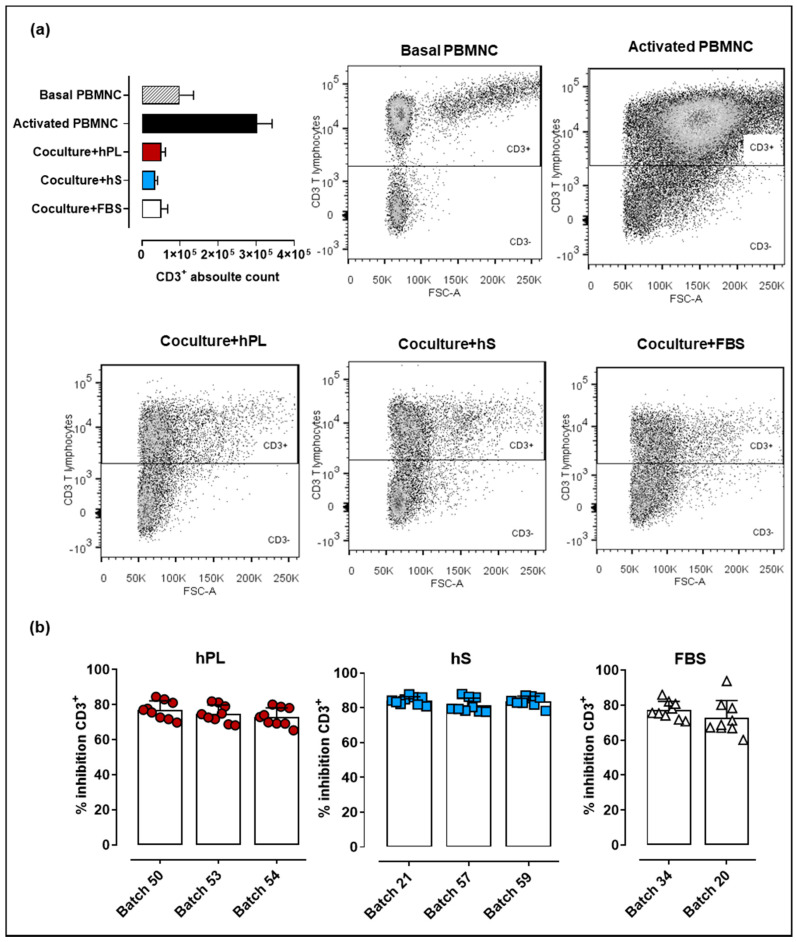
Immune suppressive potency of WJ-MSC expanded with hPL, hS and FSB (**a**) CD3^+^ absolute cell counts and corresponding flow cytometry dot plots following 72 h of peripheral blood mononuclear cells (PBMNC) culture in non-stimulated conditions (patterned gray), stimulated with anti-CD2/CD37CD28 (black) and anti-CD2/CD37CD28-activated and co-cultured with WJ-MSC expanded in hPL (RED), hS (BLUE) and FSB (WHITE); (**b**) CD3^+^ T cell inhibition (shown as %) in WJ-MSC co-cultures (*n* = 3 donors) expanded with different batches of hLP (green, *n* = 3), hS (blue, *n* = 3) and FSB (red, *n* = 2). Data is shown as mean ± SD.

Immune modulation is a key feature supporting potential therapeutic applications of WJ-MSC. In that regard, methodologies applied for cell expansion during manufacturing must ensure that immunomodulatory effect of WJ-MSC is preserved. Here we evaluated the capacity of WJ-MSC to inhibit proliferation of CD2/CD3/CD28-activated PBMNC. We observed increased levels of CD3+ cells 72-h post PBMNC activation (upper right box, Figure 6a) as compared to basal T cell proliferation in non-stimulated PBMNC (upper left box). Importantly, PBMNC co-cultured with WJ-MSC previously exposed to hPL, hS and FBS showed strong inhibition of T cell proliferation. Comparative analyses of hPL, hS and FBS indicated that PBMNC co-cultures exposed to hS-cultured WJ-MSC presented slightly higher inhibitory effect on T lymphocytes (batch “57,” 81.47 ± 4.1%, “21,” 84.09 ± 2.3% and “59,” 83.88 ± 2.8%) over hPL (*p* < 0.0001) and FBS (*p* < 0.0001) (Figure 6b). WJ-MSC expanded with hPL showed similar percentages of CD3+ cell inhibition to those observed for FBS-expanded cells (*p* > 0.05). Thus, although hS seems to enhance WJ-MSC immune inhibitory potential, hPL also supports the growth of highly potent immunomodulatory cells for cell therapy applications.

### 2.6. Analysis of Cytokines and Growth Factors Present in Culture Supernatants of WJ-MSC

**Figure 7:**
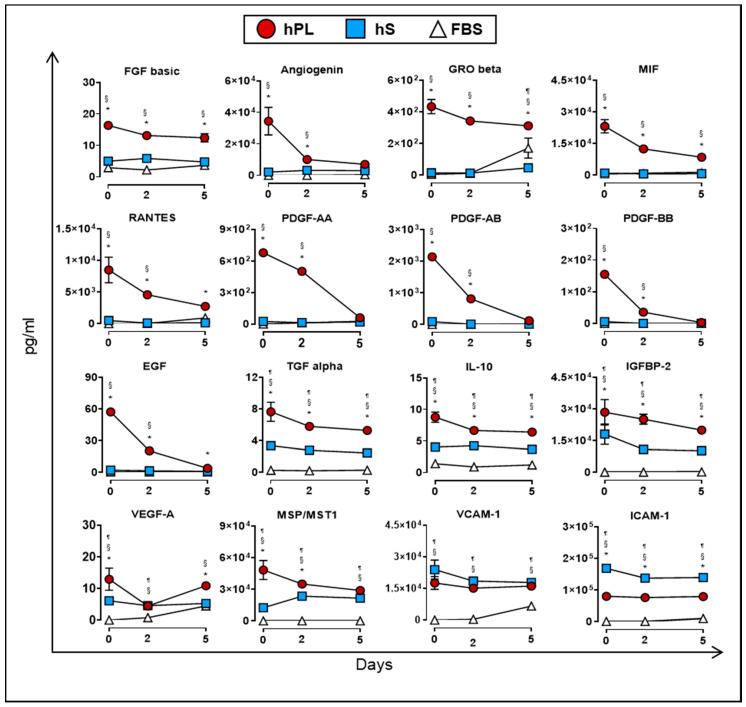
Uptake of cytokines and growth factors measured in supernatants from WJ-MSC cultured in hPL (*n* = 4), hS (*n* = 3) and FBS (*n* = 1) at days 0, 2 and 5 (*n* = 3 donors). Data are represented as mean of concentration ±SD. § *p* < 0.05 FBS vs. hS, ¶ *p* < 0.05 FBS vs. hLP, * *p* < 0.05 hLP vs. hS, as analyzed by Tukey’s test.

As previously mentioned, culture supplements provide factors that may be directly or indirectly involved in the proliferation of WJ-MSC. In this regard, we monitored the accumulation of soluble factors in supernatants from WJ-MSC expanded in the presence of hPL, hS and FBS for up to five days of culture. We first determined which factors were mainly consumed by WJ-MSC during expansion under hPL, hS and FBS supplementation. Importantly, factors uptake was mainly observed in WJ-MSC expanded with hPL but not in hS or FBS. Specifically, we found significant reduction of PDGF family AA (91.1%), AB (94.4%), BB (98%), EGF (93.2%) and Angiogenin (79.8%) in hPL-exposed WJ-MSC following 5 days of culture (as compared with Day 0, Figure 7). Furthermore, other factors strongly enriched in hPL, were also actively consumed by WJ-MSC over 5-days culture, including MSP/MST1 (40.2%), MIF (62.3%), RANTES (67.8%), TGF alpha (30.8%), IL-10 (26.9%) GRO beta (28.1%), IGFBP-2 (29.9%) and FGF basic (24.1%). Importantly, observed differences in the uptake of evaluated factors were correlated with initial high concentration in hPL as compared to starting concentrations present in hS and FBS (as shown in the Figure 2a, Figure S1). Thus, under high availability of growth factors, WJ-MSC display a clear dependency to these factors and make use of them to support active proliferation.

**Figure 8:**
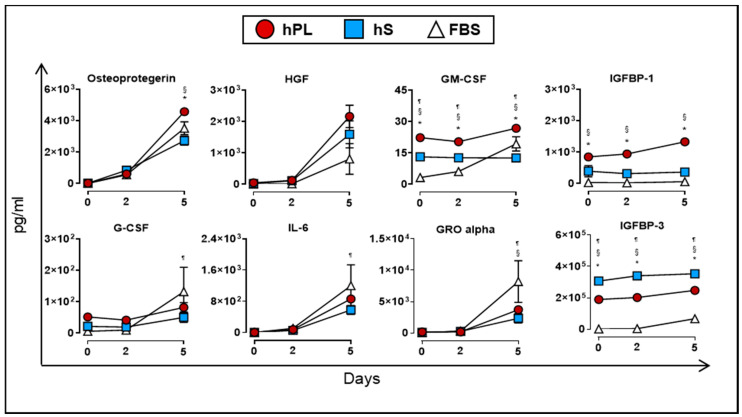
Cytokines and growth factors secreted by WJ-MSC (*n* = 3 donors) cultured in hPL (*n* = 4), hS (*n* = 3) and FBS (*n* = 1) and detected in supernatants at day 0, 2 and 5. Data are represented as mean of concentration ± SD. * *p* < 0.05 hLP vs. hS, ¶ *p* < 0.05 FBS vs. hLP and § *p* < 0.05 FBS vs. hS, analyzed by Tukey’s test.

Finally, we evaluated the production of soluble factors by WJ-MSC when cultured in the presence of hPL, hS and FBS. At day 5, we detected significant accumulation of osteoprotegerin, HGF, GM-CSF and IGFBP-1 in supernatants from WJ-MSC cultured in hPL as compared to hS and FBS (*p* < 0.05, Figure 8). Additional immunomodulatory factors such as IL-6 (266.5-fold at day 5 vs. day 0), GRO alpha (24.3-fold at day5 vs. 0) and G-CSF (6.03-fold at day 5 vs. day 0) also showed significant enrichment in hPL-supplemented WJ-MSC, although we did not find significant differences with hS and FBS. Interestingly, VEGF-A showed a transient uptake of 8.4% after 2 days of culture by WJ-MSC in hPL (*p* < 0.0001) followed by active accumulation up to 5 days of culture (2.4-fold vs. day 2, *p* < 0.0001, Figure 7). Taken together, we demonstrated that hPL is enriched with several critical growth factors that are actively taken by WJ-MSC to support cell expansion. These factors might be closely linked to the induction of proliferation signals in WJ-MSC that are absent in hS and FBS. Importantly, hPL also enhanced the release of several growth factors that are involved in differentiation and immune modulation processes functions driven by -WJ-MSC.

## 3. Discussion

In the present study, we introduced a methodology to generate highly homogeneous hPL batches intended for the expansion of human WJ-MSC in the perspective of improving manufacturing scaling of cell therapies. We strongly focused on the characterization of hPL composition regarding soluble factor content and stability and performed comprehensive analyses to determine critical molecules that impact expansion, survival, differentiation, and immunomodulatory potential of WJ-MSC. Finally, we were able to demonstrate that hPL preparations display a unique profile of factors that are actively taken up by WJ-MSC to support efficient cell expansion while preserving their biological and potential therapeutic features. Advanced Therapy Medicinal Products (ATMP) based on WJ-MSC are currently being under intense development as cell therapeutics due to their regenerative characteristics, potent immunomodulatory properties, low risk for carrying somatic mutations, demonstrated effectivity and absence of adverse effects in the clinical setting, making them a promising therapeutic resource for the treatment of several pathologies involving regeneration of tissue injuries in several systems (neural, myocardial, skin, liver, kidney, cartilage, bone, muscle) [18,19]. Production of MSC-based cellular therapies for clinical use requires the standardization of critical methods for isolation, expansion and conservation of WJ-MSC under conditions that comply with standards of good manufacturing practices (GMP), hereby ensuring proper identity, purity, viability, stability and quantity parameters of the cell compound present in the final product. In that regard, one of the biggest challenges facing ATMP manufacturers involves setting up cell expansion methodologies that allow procuring sufficient amounts of highly homogenous, viable and stable cell batches that comply with required doses (ranging from 0.5 × 10^6^ to 4 × 10^7^ cells/kg body weight) for clinical use [20]. Thus, large-scale manufacturing bioprocess must be carried out in the presence of culture supplements that enhance cell growth, display biosafety features (xenogeneic free) and preserve or even enhance the intended therapeutic effect of WJ-MSC.

It is assumed that the presence of soluble factors in culture supplements favors the optimal proliferation of the MSC-based product allowing the production of large batches that could cover a broad spectrum of therapeutic applications. Here we evaluated representative soluble factors of the platelet secretome that might be exploited as culture supplements in hPL preparations. hPL-pools showed enhanced content of a wide variety of soluble factors when compared to factor profiles present in hS or standard FBS. Identified factors here, belong to the group of immunomodulatory molecules (MIF, IL-10, IL-6, TGF alpha, GM-CSF, G-CSF), chemokines (RANTES, GRO alpha and Beta) and pro-angiogenic and pro-survival factors (IGFBP-1 and 2, VEGF-A, PDFG-AA, AB and BB, FGF basic, EGF, MSP/MST1, HGF and osteoprotegerin). Consistent with previous studies, VEGF-A, TGF, FGF, EGF, RANTES, IL-6 and PDGF were significantly higher in hPL than in FBS [16,21,22,23]. Moreover, the secretome profile here obtained have been also recognized to contain critical cytokines and growth factors that promote angiogenic processes, cell repair and macrophage activation leading to wound healing [9,10]. In fact, factors such as PDGF-AB, EGF and TGF beta are involved in major cellular processes including proliferation, differentiation, migration and apoptosis, thus contributing to the renewal of different cell types [24]. Not surprisingly, these supplements have been extensively used in regenerative medicine, maxillofacial surgery and orthopedics by contributing to the recovery of injured tissues [11].

Of note, soluble factor profiling obtained in this study allowed us to further distinguish interesting usage patterns of factors such as PDGF family members AA, AB, BB, EGF, Angiogenin, RANTES, TGF alpha, IL-10, GRO beta, IGFBP-2, FGF basic by WJ-MSC in culture, which strongly correlated with enhanced cell expansion. Similar findings were described for PDGF-BB, RANTES and EGF which were significantly used by bone marrow, umbilical cord and adipose tissue-derived MSC in the presence of hPL [22]. In our study, neither hS nor FBS did show striking profiles of growth factor consumption, pointing to hPL as suitable source of critical factors to support WJ-MSC in the context of cell expansion. Noteworthily, PDGF and EGF were reported to influence the expression of cell cycle-associated genes such as cMyc and KLF4 during MSC expansion [22,25,26,27,28]. Furthermore, TGF alpha, HGF and EGF augmented cell survival and PDGF-BB reduced cell loss induced by apoptosis [16]. Among pivotal genes for the control of cell cycle homeostasis, CDK4 and the DNA repair gene Pold3 were strongly induced in MSC cultures treated with a combination of FGF-2 and EGF [28]. Thus, cumulative evidence points FGF basic, PDGF-BB and EGF contained in hPL, as pivotal factors to stimulate proliferation rates and cell doubling numbers without inducing early senescence in MSC. Here we demonstrated that additional factors present in hPL supplement such as RANTES, TGF alpha, IL-10, GRO beta and IGFBP-2 might be further required to improve proliferation, viability, stability and potency of WJ-MSC and therefore constitutes an essential raw material used for the manufacture of WJ-MSC-based ATMP.

Taking into account the essential role of hPL as major source for growth-promoting factors needed by WJ-MSC expansion, we also assessed the capacity of hPL to induce secretory responses by WJ-MSC during expansion phases that could intervene in biological and therapeutic functions but also ensure compliance of those quality criteria established for optimal batch release. Accordingly, we observed significant production of factors such as osteoprotegerin, HGF, IL-6, GRO alpha and VEGF-A following 5 days of expansion of WJ-MSC in the presence of hPL but not in hS or FBS. Some of these factors have been previously correlated with the production of immunoregulatory signals that downsize the immune response [29], facilitate efficient cell engraftment and support wound healing [30]. Moreover, our report is consistent with previous observations pointing to VEGF-A, HGF, GRO family, osteoprotegerin and IL-6 secretion by hPL-expanded MSC as crucial signals in promoting angiogenesis and formation of reparative M2 macrophages [22,31]. Specifically, WJ-MSC were showed to be capable of producing secretory signals depending on the microenvironment in which they were grown and promoted anti-inflammatory responses by activating the induction of PGE2, IDO, TGF beta and HGF, thereby contributing to suppression of T cell proliferation and induction of Treg lymphocytes [32]. Altogether, hPL has consistently showed a beneficial effect on preserving MSC biology during cell expansion but also has proven to enhance biological features that are pivotal for their therapeutic effect in potential clinical scenarios. Our study has further confirmed that highly reproducible hPL batches can actively induce the production of soluble mediators by WJ-MSC.

Human blood components have become a feasible alternative for cell growth supplements and have gained acceptability over the last decade specially because of its ability to strongly support cell proliferation. Here we observed that WJ-MSC expanded in hPL enhanced their proliferative capacity, observing an increase of 33.3% in PD and consequent reduction of PDT (by 38.1%), as compared to WJ-MSC expanded in hS or FBS. Several studies described mixed proliferation rates of different MSC types following hPL supplementation [13,22,31,33]. In particular Kandoi et al. evidenced significantly faster cell growth of umbilical cord tissue-derived MSC in hPL-enriched cultures, reaching PDT of 20.95 h vs. PDT in FBS [5]. In contrast, Tancharoen et al. revealed that amniotic fluid-derived MSC cultured in 10% FBS or hPL did not show differences in cell number and shared a fibroblast-like morphology [34]. In that regard, cell morphology has evolved as critical parameter predicting the expansion response to a particular cell supplement. In fact, we observed clear differences in the morphology of the MSC cultured in hPL as compared to hS and FBS, the last presenting larger cytoplasm than cells cultured with hPL. These findings were further correlated as shown by Fernandez et al., where less elongated cell morphology in MSC grown in the presence of fetal calf serum (FCS), was correlated with significantly higher number of focal adhesions covering larger growth areas and higher tension forces identified, that were not present in hPL-expanded cells [33].

Further parameters such as stability and reproducibility should be taken in account when the feasibility in the use of hPL is assessed, considering the fact that hPL constitutes a critical row material for manufacturing process under GMP. Here we demonstrated that batches derived from LLP-pools displayed very low inter-batch variation when cytokines and growth factors were evaluated, even under different storage conditions (−80 °C, 4 °C and room temperature). Accordingly, Mohamed et al. observed that hPL was stable for up to 15 months at −20 °C as shown for specific factors such as IGF-1, TGF beta and VEGF [21]. Similar quality criteria for suitable hPL was also applied for PDGF-AB, EGF and basic-FGF levels, identifying hPL but not hS [35]. Additional quality parameters of hPL preparations regarding its impact on critical biological attributes of WJ-MSC should be also considered. For instance, we did not observed alterations in the immunophenotypic profile of WJ-MSC and their immunosuppressive action on T lymphocytes, when cultured in the presence of hPL, hS and FBS. Moreover, hPL-cultured WJ-MSC maintained an expected differentiation profile towards osteogenic, adipogenic, chondrogenic lineages and preserved genetic stability. These results are in agree with previous observations where some of these parameters were assessed in MSC from other origins during culture protocols applying hPL [5,6,7]. On the other hand, it has been reported that certain soluble factors present in hPL influence the differentiation of MSC such as TGF beta which intervenes in cell proliferation and induces differentiation to the chondrogenic lineage [16], FGF-2 and PDGF-BB both involved in the expression of PPAR-Υ and Runx-2 genes involved in enhanced adipogenesis and osteogenesis, respectively [28]. Therefore, hPL-derived factors are widely recognized to promote differentiation of MSC, thus favoring the potential of these cells to be used in regeneration processes and physiological remodeling [36].

There is still a lack of consensus regarding the introduction of unified safety criteria for the manufacture and release of hPL batches intended for WJ-MSC-based ATMP production. Among them, pathogen safe becomes a relevant issue as LLP pooling increases the risk of pathogen transmission. Therefore, the inclusion of techniques that lead to pathogen inactivation without altering the characteristics of hPL is vital to produce GMP grade supplements. Recently, protocols for pathogen inactivation including solvent/detergent, photoinactivation or nanofiltration technology have been tested, proving to be innocuous for the prepared hPL in terms of factors composition and mechanism of action [12,13]. In that regard, one of the limitations of the present study deals with the absence of methodologies for pathogen inactivation and their impact on the stability of soluble factors present in hPL batches and the biological activity of WJ-MSC. Such safety criteria is of vital importance when these supplements will be used for the manufacture of ATMPs. Nevertheless, we anticipate future reports introducing better technologies for hPL treatment that will efficiently lead to effective viral inactivation without altering the valuable biological properties of hPL. Furthermore, additional research focused on providing robust data for setting up production and quality criteria for GMP-grade hPL should be established, in order to introduce standard methodologies for batch production focused on the preservation of safety and essential biological properties of hPL which are linked to tissue regeneration, cell survival and stability. Finally, additional biological mechanisms by which hPL supports MSC properties should be further explored, including the interesting role of platelet-derived extracellular vesicles and their impact on overall cellular MSC gene expression, differentiation and survival.

In conclusion, this study provides additional hints that support the efficacy of hPL as an alternative culture supplement for the expansion of WJ-MSC in where specific platelet-derived factors play a key role. Thanks to its highly bioactive secretome, hPL promotes an optimal microenvironment for the proliferation of WJ-MSC that favors increased cellular yields, superior viability and secretion of immunomodulatory, pro-angiogenic and cell survival signals that ultimately will potentiate the immune therapeutic and regenerative characteristics of WJ-MSC-based therapies.

## 4. Materials and Methods

### 4.1. Human Platelet Lysate (hPL) Production

Production of hPL was carried out from leukocyte-poor platelet (LPP) bags procured from O+ blood donors at IDCBIS blood bank. The process for LPP obtention was based on buffy coat separation performed in whole blood bags. Each batch of hPL consisted in LPP pools from three different donors, which were further adjusted to a concentration of 6 × 10^5^ platelets/µL with 1 × phosphate-buffered saline (PBS) (Gibco, Life Technologies, Carlsbad, CA, USA). Platelet lysis was subsequently performed by two cycles of freezing (−80 °C)/thawing (37 °C) and finally stored in 30 mL aliquots. Direct after thawing, each hPL aliquot was centrifuged (4000× *g* × 10 min) to remove cellular debris and filtered through 0.2 μm before use as culture supplement in a 10% final concentration (Figure 1).

### 4.2. Human SERUM (hS) Production

To produce human serum, whole blood bags from O+ blood donors were subjected to buffy coat separation by centrifugation at 4100 rpm × 7 min, plasma was recovered in additional bags and frozen. Plasma bags were thawed with constant agitation at room temperature and subsequently added calcium chloride (Sigma-Aldrich, Merck, Germany) at 20% (w/v) and incubated at 4 °C overnight in constant agitation. Serum-converted plasma was recovered by centrifugation (4000× *g* × 5 min), aliquoted and stored at −80 °C. HS aliquots were filtered through 0.2 μm (Figure 1) prior use as a culture supplement in a final concentration of 10%.

### 4.3. Isolation and Culture of WJ-MSC

Umbilical cord (UC) samples were collected from full-term births (caesarian and vaginal deliveries). The procurement of umbilical cord tissue for WJ-MSC isolation and cryopreservation was approved by the institutional ethical board at Secretaría Distrital de Salud, Bogotá under the acceptance reference 2019EE44993. Informed consent was applied to donors before donation. UC tissues were cut into 3 cm pieces and washed in 0.9% saline with 1% penicillin/streptomycin 10,000 U/mL (Gibco, Life Technologies, Carlsbad, CA, USA). Next, each fragment was slit longitudinally, arteries and vein were removed and dismissed and the Wharton’s jelly was separated and directly seeded in 75-cm^2^ culture flasks containing Dulbecco’s Modified Eagle’s medium (DMEM) with low glucose (Gibco, Life Technologies, Carlsbad, CA, USA), 1% penicillin/streptomycin 10,000 U/mL and supplemented with 10% of fetal bovine serum (FBS), hS or hPL plus 8 IU/mL of heparin. WJ-MSC cultures were maintained at 37 °C in a humidified atmosphere with 5% CO_2_. Culture media was replenished every 3rd to 4th day. Confluent cultures (80%) were harvested by using 0.25% trypsin-EDTA, (Gibco, Life Technologies, Carlsbad, CA, USA) followed by cell count. For WJ-MSC expansion, 1500 to 2500 cells/cm^2^ were used. Cell cryopreservation was carried out at 80% confluence in 60% DMEM, 30% hPL and 10% DMSO and finally stored at −196 °C.

### 4.4. Evaluation of Cell Growth Kinetics in hPL, hS and FBS-Supplemented Media

The growth kinetics of WJ-MSC (*n* = 4) was evaluated in cell cultures supplemented with 10% hPL (*n* = 3, batch 2, 5, 8), 10% hS (*n* = 3, batch 74, 77, 84) and 10% FBS (*n* = 2, Batch 1, 2). Briefly, 2000 cells/cm^2^ WJ-MSC were seeded in 24-well culture plates with DMEM (Gibco, Life Technologies, Carlsbad, CA, USA) and tested media supplement. Cultures were maintained at 37 °C in a humidified atmosphere with 5% CO_2_ and number of viable cells in culture were determined every 24 h for 5 days. Cell viability was assessed by CellTiter-Glo Luminescent Cell Viability Assay (Promega, Wisconsin, USA) following manufacturer’s instructions. Briefly, culture supernatants were removed and 200µL of DMEM and 200 µL of CellTiter-Glo per well were added. Next, the well-plate was incubated for 2 min with constant agitation to allow interaction between the ATP produced by cells and the substrate luciferin catalyzed by Ultra-GLO rLuciferase, generating luminescent signal. Luminescence was further measured in a plate reader (Synergy/HTX, BioTek, Winooski, Vermont, USA). The number of viable cells present in each well evaluated was proportional to the luminescent signal recorded. Furthermore, population doubling (PD) during 5-day culture was also determined using the following formula [1]:

(1) $$PD=\frac{ln\left(\frac{N}{N_{0}}\right)}{ln\;\left(2\right)}$$

where *N*_0_ is the luminescent signal obtained after 24 h of culture and *N* is the luminescent signal obtained after 5 days of culture.

Similarly, population doubling time (*PDT*) was obtained using the formula:

(2) $$PDT=\frac{D}{PD}$$

where *D* is the days of cultivation and *PD* is the population doubling.

### 4.5. WJ-MSC Immunophenotype Analyses

Expanded WJ-MSC (*n* = 3) supplemented with 10% hPL (*n* = 3, batch 50, 53, 54), hS (*n* = 3, batch 21, 54, 57) and FBS (*n* = 2, batch 1, 2) (Gibco, Life Technologies, Carlsbad, CA, USA) were characterized immunophenotypically using antibodies against the following human antigens—CD90-APC, CD73-PE/Cy7, CD105-PE, CD274-PE, CD45-APC/Cy7, CD34-PerCP-Cy5.5, CD31-PE and HLA-DR-Pacific Blue (Biolegend, San Diego, USA). Appropriate isotype controls for each of the antibodies were used. Cells cultures a 70–80% confluence were harvested using 0.25% trypsin-EDTA, (Gibco, Life Technologies, Carlsbad, CA, USA) and centrifuged at 1200 rpm × 6 min. Cell pellets were resuspended in 100 µL of 1× PBX with 5% Bovine Serum Albumin (Gibco, Life Technologies, Carlsbad, CA, USA) containing the corresponding antibody. Cell suspension was incubated for 30 min at 4 °C, washed with 1X PBS and resuspended in 200 µL 1× PBS. Flow cytometry analysis were carried out with a FACSCanto II™ instrument (BD, Franklin Lakes, NJ, USA) and data were analyzed with the FlowJo vX.7.0 software package (TreeStar, USA).

### 4.6. Assessment T Lymphocyte Suppression by WJ-MSC

For immune-modulatory potency of WJ-MSC, cells (*n* = 3) were expanded in DMEM supplemented with 10% (*n* = 3, batch 50, 53, 54), hS (*n* = 3, batch 21, 54, 57) or FBS (*n* = 2, batch 1, 2) and evaluated for their ability to inhibit CD3+ lymphocytes. Briefly, 70–80% confluent WJ-MSC were co-cultured with peripheral blood mononuclear cells (PBMNC) in a ratio of 5 × 10^4^ WJ-MSC per well in 24-well plates at 37 °C, 5% CO_2_ for 24 h. PBMNC were isolated by Ficoll Paque (GE Healthcare) density gradient separation and cells (5 × 10^5^/well) were added to cultured WJ-MSC in the presence of anti-CD2/CD3/CD28-conjugated beads (Miltenyi Biotec GmbH, Bergisch Gladbach, Germany). Basal non-stimulated and anti-CD2/CD3/CD28-stimulated PBMNC were used as controls. Activated PBMNC-WJ-MSC co-cultures were incubated for 72 h at 37 °C in 5% CO_2_. Inhibitory effect of WJ-MSC on lymphocyte proliferation was determined by CD3+ absolute cells counts in a flow cytometer (FACSCanto II™, BD, Franklin Lakes, NJ, USA). Data were analyzed with FlowJo vX.7.0 software package (TreeStar, USA). Results are reported as the mean of the percentage of T lymphocyte inhibition calculated as follows [37]:

(3) Absolute proliferation of T lymphocyte = stimulated PBMNC−non stimulated PBMNC 

(4) $$\text{co-culture}\;T\;\mathrm{lymphocyte}\;\mathrm{Proliferation}\;\left(\%\right)\;=\;\left(\frac{\mathrm{Proliferation}\;\mathrm{in}\;\text{co-culture}}{\mathrm{Absolute}\;\mathrm{proliferation}\;\mathrm{of}\;T\;\mathrm{lymphocyte}\;}\right)*100$$

(5) T lymphocyte Inhibition (%) = 100− co-culture T lymphocyte Proliferation(%)

### 4.7. Determination of Cytokine and Growth Factor Levels by Multiplex Bead Analysis

Production of cytokines and growth factors contained in culture supernatants and media supplements were analyzed by Luminex Human Magnetic Assay (R & D Systems, Abingdon, UK). Factors analyzed were—Angiogenin, RANTES, Growth-regulated Oncogene protein alpha and beta (GRO alpha y GRO beta), Epidermal Growth Factor (EGF), basic Fibroblast Growth Factor (FGF basic), Granulocyte Colony-Stimulating Factor (G-CSF), Granulocyte Macrophage Colony-Stimulating Factor (GM-CSF), Hepatocyte Growth Factor (HGF), Intercellular Adhesion Molecule 1 (ICAM-1), Vascular Cell Adhesion Molecule 1 (VCAM-1), Insulin-like Growth Factor Binding Proteins (IGFBP)-1, IGFBP-2, IGFBP-3, Interleukin IL-10, IL-6, Macrophage Migration Inhibitory factor (MIF), Macrophage-Stimulating Protein (MSP/MST1), Platelet Derived Growth Factor (PDGF)-AA, PDGF-BB, PDGF-AB, Transforming Growth factor alpha (TGF-alpha), Osteoprotegerin and Vascular Endothelial Growth Factor (VEGF-A). For culture supplement analyses, hPL (*n* = 3), hS (*n* = 3) and FBS (*n* = 3) batches were diluted 1:10 and directly evaluated by multiplex cytokine assay. Similarly, for hPL stability tests, hPL batches stored at −80 °C for more than 30 days (*n* = 9), batches kept at room temperature for 5 days (*n* = 9) and batches stored at 4 °C for up to 30 days (*n* = 9) were diluted (1:10) and evaluated as previously described. Finally, cytokines and growth factors present in supernatants of cultured WJ-MSC (*n* = 4) expanded in the presence of hPL (*n* = 4), hS (*n* = 3) and FBS (*n* = 1) were analyzed. In short, WJ-MSC were seeded in a density of 2000 cells/cm^2^ and supernatants were collected after 48 h and 120 h of culture. Fresh culture medium was collected from day zero. Multiplex cytokine assay was performed according to manufacturer’s instructions. Fluorescence signals were detected using the Luminex 200TM detection system (Invitrogen™, Carlsbad, California USA,) and data were analyzed in the xPONENT Software. Concentrations are presented in pg/mL.

### 4.8. Statistical Analysis

Statistical analysis of the data were performed by two-way ANOVA and comparison between groups was carried out by Tukey’s test, using GraphPad Prism software version 8.0 (La Jolla, CA, USA). Data is displayed as mean ± standard deviation. Statistical significances were determined using *p* < 0.05. The data of cytokines and growth factors present in the supplements were analyzed in the ClustVis web program using the Heatmap and Principal Component Analysis (PCA).

## Figures and Tables

**Table 1:** Characterization of cellular populations (platelets, erythrocytes, leukocytes) and corresponding pH values of different hPL batches from LPP pools (*n* = 3 donors, O^+^).

Batch	Platelets/µL		Leukocytes/µL		Erythrocytes/µL		pH
	Initial	Adjusted	Initial	Adjusted	Initial	Adjusted	Final
2	9.7 × 10^5^	6.1 × 10^5^	1.0 × 10^2^	6.0 × 10^1^	5.0 × 10^4^	3.0 × 10^4^	*
5	8.3 × 10^5^	5.8 × 10^5^	5.9 × 10^2^	4.0 × 10^2^	4.0 × 10^4^	3.0 × 10^4^	*
6	1.1 × 10^6^	5.8 × 10^5^	2.9 × 10^2^	1.8 × 10^2^	8.0 × 10^4^	4.0 × 10^4^	*
8	6.1 × 10^5^	5.9 × 10^5^	1.4 × 10^2^	1.3 × 10^2^	3.0 × 10^4^	3.0 × 10^4^	*
23	1.1 × 10^6^	5.8 × 10^5^	1.2 × 10^2^	7.0 × 10^1^	5.0 × 10^4^	2.0 × 10^4^	7.25
25	7.8 × 10^5^	6.0 × 10^5^	1.2 × 10^2^	7.0 × 10^1^	2.0 × 10^4^	1.0 × 10^4^	7.66
26	1.1 × 10^6^	5.8 × 10^5^	2.3 × 10^2^	1.3 × 10^2^	6.0 × 10^4^	3.0 × 10^4^	7.21
27	6.9 × 10^5^	6.1 × 10^5^	2.6 × 10^2^	2.2 × 10^2^	3.0 × 10^4^	2.0 × 10^4^	7.53
28	1.1 × 10^6^	5.8 × 10^5^	3.5 × 10^2^	1.8 × 10^2^	3.0 × 10^4^	2.0 × 10^4^	7.50
29	1.1 × 10^6^	5.9 × 10^5^	1.5 × 10^2^	8.0 × 10^1^	5.0 × 10^4^	3.0 × 10^4^	7.25
30	8.0 × 10^5^	6.0 × 10^5^	1.1 × 10^2^	8.0 × 10^1^	3.0 × 10^4^	2.0 × 10^4^	7.63
31	8.5 × 10^5^	5.9 × 10^5^	2.5 × 10^2^	1.8 × 10^2^	3.0 × 10^4^	2.0 × 10^4^	7.54
33	1.3 × 10^6^	5.9 × 10^5^	2.5 × 10^2^	1.5 × 10^2^	1.9 × 10^5^	9.0 × 10^4^	6.04
50	1.5 × 10^6^	6.4 × 10^5^	1.7 × 10^2^	7.0 × 10^1^	7.0 × 10^4^	3.0 × 10^4^	7.20
53	1.0 × 10^6^	6.0 × 10^5^	5.0 × 10^1^	3.0 × 10^1^	4.0 × 10^4^	2.0 × 10^4^	*
54	1.4 × 10^6^	5.7 × 10^5^	7.0 × 10^1^	3.0 × 10^1^	2.2 × 10^5^	9.0 × 10^4^	*
Mean	10 × 10^5^	5.9 × 10^5^	2.0 × 10^2^	1.3 × 10^2^	6.4 × 10^4^	3.3 × 10^4^	7
SD	2.5 × 10^5^	1.6 × 10^4^	1.3 × 10^2^	9.3 × 10^1^	5.8 × 10^4^	2.3 × 10^4^	0.5

* Data not available, SD: standard deviation.

## Supplementary Materials

The following are available online at https://www.mdpi.com/1422-0067/21/17/6284/s1.

## Abbreviations

WJ-MSC	Wharton’s Jelly Mesenchymal Stromal Cells
hPL	Human Platelet Lysate
hS	Human Serum
FBS	Fetal bovine serum
ATMP	Advanced Therapy Medicinal Products
