# Supplementary material for: Human Platelet Lysate Supports Efficient Expansion and Stability of Wharton’s Jelly Mesenchymal Stromal Cells via Active Uptake and Release of Soluble Regenerative Factors
Source: Int J Mol Sci. 2020 Aug 31;21(17):6284. doi: 10.3390/ijms21176284 (PMC7503902; doi:10.3390/ijms21176284)
Supplement: Supplementary file 1 [file ijms-21-06284-s001.zip › suplementary Material.pdf]

TABLE S1

Table S1: Cytokines and growth factors present in culture supplements derived from blood components (hLP, hS and FSB). n = 3, mean ± SD

Factors	hLP (pg/ml)	hS (pg/ml)	FSB (pg/ml)
**Angiogenin**	$1.3x10^5 \pm 4.5x10^4$	$9.0x10^3 \pm 4.4x10^3$	7.2 ± 3.2
**CCL5/RANTES**	$8.6x10^4 \pm 1.3x10^4$	$2.9x10^3 \pm 1.1x10^3$	0.3 ± 0.3
**CXCL2/GROβ**	$2.4x10^3 \pm 2.1x10^2$	23.8 ± 10.0	4.7 ± 0.0
**CXCL1/GROα**	$9.9x10^4 \pm 5.5x10^2$	$5.5x10^2 \pm 1.5x10^2$	$2.3x10^2 \pm 2.0x10^2$
**EGF**	$4.0x10^2 \pm 32.44$	9.0 ± 3.7	2.5 ± 0.9
**FGF Basic**	$4.3x10^2 \pm 28.50$	22.0 ± 8.8	2.2 ± 0.6
**G-CSF**	$1.7x10^3 \pm 1.4x10^2$	92.9 ± 36.0	7.2 ± 6.2
**GM-CSF**	$5.4x10^2 \pm 50.9$	63.3 ± 29.2	3.4 ± 3.0
**HGF**	$4.8x10^2 \pm 33.4$	$1.4x10^2 \pm 46.58$	1.2 ± 2.1
**ICAM-1**	$8.2x10^5 \pm 1.2x10^5$	$1.5x10^6 \pm 2.5x10^5$	$2.5x10^3 \pm 8.6x10^2$
**IGFBP-1**	$9.5x10^4 \pm 6.2x10^2$	$2.0x10^3 \pm 1.6x10^3$	$1.9x10^2 \pm 0.0$
**IGFBP-2**	$1.9x10^5 \pm 1.2x10^5$	$3.7x10^4 \pm 3.8x10^4$	28.6 ± 24.8
**IGFBP-3**	$7.3x10^5 \pm 1.1x10^5$	$2.0x10^6 \pm 2.3x10^5$	$6.6x10^3 \pm 1.7x10^3$
**IL-6**	43.36 ± 4.8	6.6 ± 3.2	2.3 ± 0.0
**IL-10**	$2.8x10^2 \pm 19.5$	21.0 ± 7.5	6.0 ± 1.1
**MIF**	$1.1x10^5 \pm 1.9x10^4$	$1.8x10^3 \pm 3.5x10^2$	$2.5\ x10^2 \pm 20.8$
**MSP/MST1**	$4.2x10^5 \pm 4.3x10^4$	$1.4x10^5 \pm 4.2x10^4$	80.8 ± 48.3
**PDFG-BB**	$1.4x10^4 \pm 1.5x10^3$	29.1 ± 11.5	0.1 ± 0.1
**PDFG-AA**	$3.7x10^3 \pm 5.0x10^2$	$1.8x10^2 \pm 45.4$	2.7 ± 1.1
**TGFα**	$1.4x10^2 \pm 24.3$	14.3 ± 14.1	4.2 ± 1.3
**Osteoprotegerin**	$3.9\ x10^2 \pm 1.2x10^2$	$2.1x10^2 \pm 419$	2.3 ± 2.0
**PDGF AB**	$5.7x10^3 \pm 6.7x10^2$	$3.4x10^2 \pm 83.4$	12.8 ± 11.1
**VCAM-1**	$2.8x10^5 \pm 1.0x10^5$	$2.4x10^5 \pm 7.5x10^4$	$5.5x10^2 \pm 0.0$
**VEGF-A**	90.13 ± 20.6	20.1 ± 7.6	0.4 ± 0.7

TABLE S2

Table S2: Karyotype evaluation of the different WJ-MSC donors belonging to the Master Bank of WJ-MSC

Donor	Passage	G Banding karyotype	Interpretation
WJ-MSC 180	Pase 2	46XY	No numeric or structural alteration
WJ-MSC 182	Pase 1	46XY	No numeric or structural alteration
WJ-MSC 190	Pase 1	46XY	No numeric or structural alteration
WJ-MSC 181	Pase 1	46XY	No numeric or structural alteration
WJ-MSC 195	Pase 1	46XY	No numeric or structural alteration
WJ-MSC 197	Pase 1	46XY	No numeric or structural alteration
WJ-MSC 198	Pase 1	46XY	No numeric or structural alteration
WJ-MSC 189	Pase 1	46XY	No numeric or structural alteration
WJ-MSC 191	Pase 2	46XY	No numeric or structural alteration
WJ-MSC 206	Pase 1	46XY	No numeric or structural alteration
WJ-MSC 200	Pase 1	46XY	No numeric or structural alteration
WJ-MSC 203	Pase 1	46XX	No numeric or structural alteration
WJ-MSC 205	Pase 1	46XX	No numeric or structural alteration
WJ-MSC 196	Pase 1	46XX	No numeric or structural alteration
WJ-MSC 199	Pase 1	46XX	No numeric or structural alteration
WJ-MSC 202	Pase 1	46XY	No numeric or structural alteration

FIGURE S1

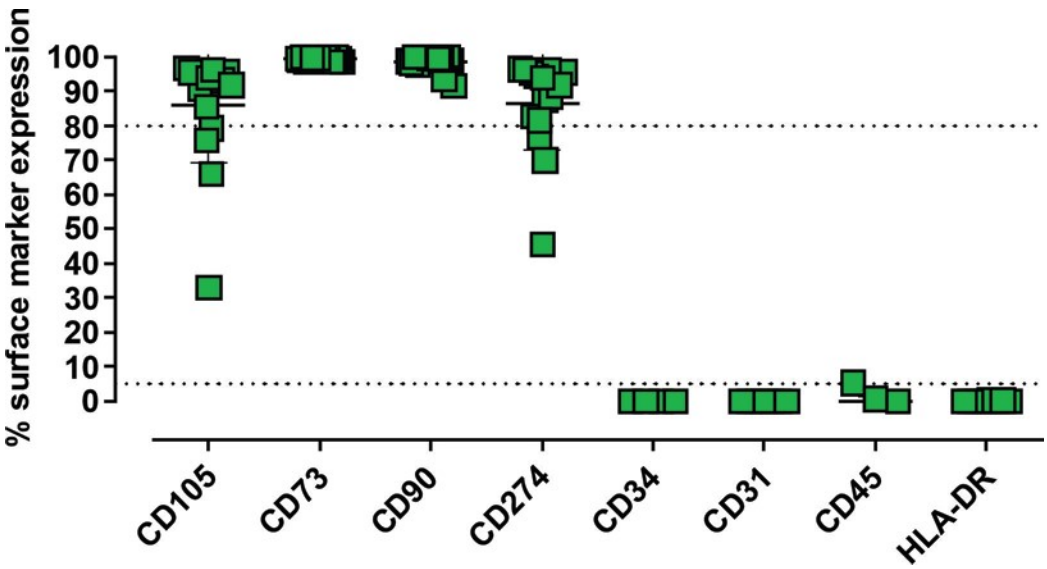

Figure S1: Immunophenotypic profile of WJ-MSC belonging to the Master Cell Bank. Data showed as expression percentage of surface markers; n = 16, mean±SD, *p<0,05 analyze by Tukey's test.

FIGURE S2

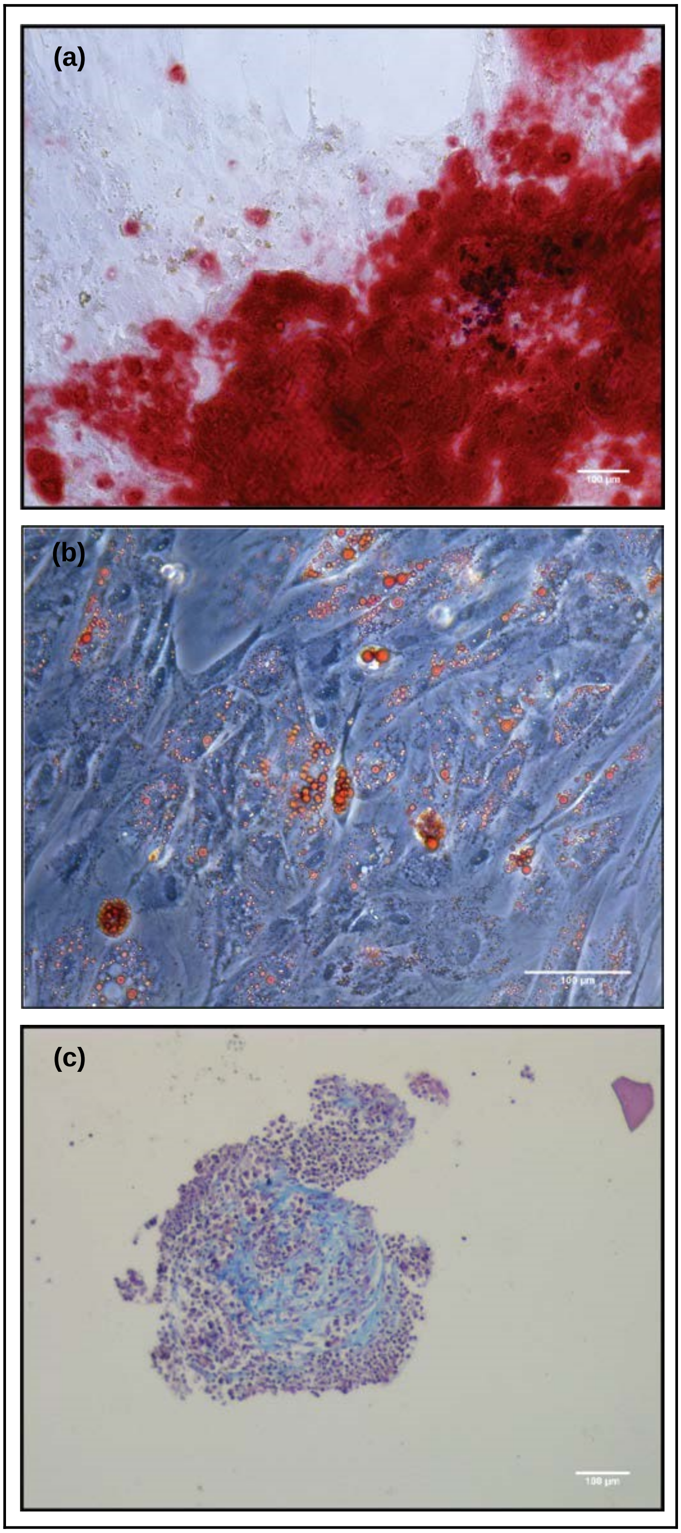

Figure S2: Evaluation of the differentiation potential of WJ-MSC. (a) Differentiation to the osteoblast lineage, red coloration characteristic of calcium deposits (staining with alizarin red), Magnification 100X, (b) Differentiation to the adipogenic lineage, red coloration characteristic of fat vacuoles (staining with oil Red), Magnification 200X, (c) Differentiation to the chondrogenic lineage, coloration blue characteristic of type I collagen matrix (staining with Masson's trichrome), Magnification 100X. Representation of one donor of WJ-MSC.
