# Supplementary figures and images for: Human Platelet Lysate Supports Efficient Expansion and Stability of Wharton’s Jelly Mesenchymal Stromal Cells via Active Uptake and Release of Soluble Regenerative Factors
Source: Int J Mol Sci. 2020 Aug 31;21(17):6284. doi: 10.3390/ijms21176284 (PMC7503902; doi:10.3390/ijms21176284)

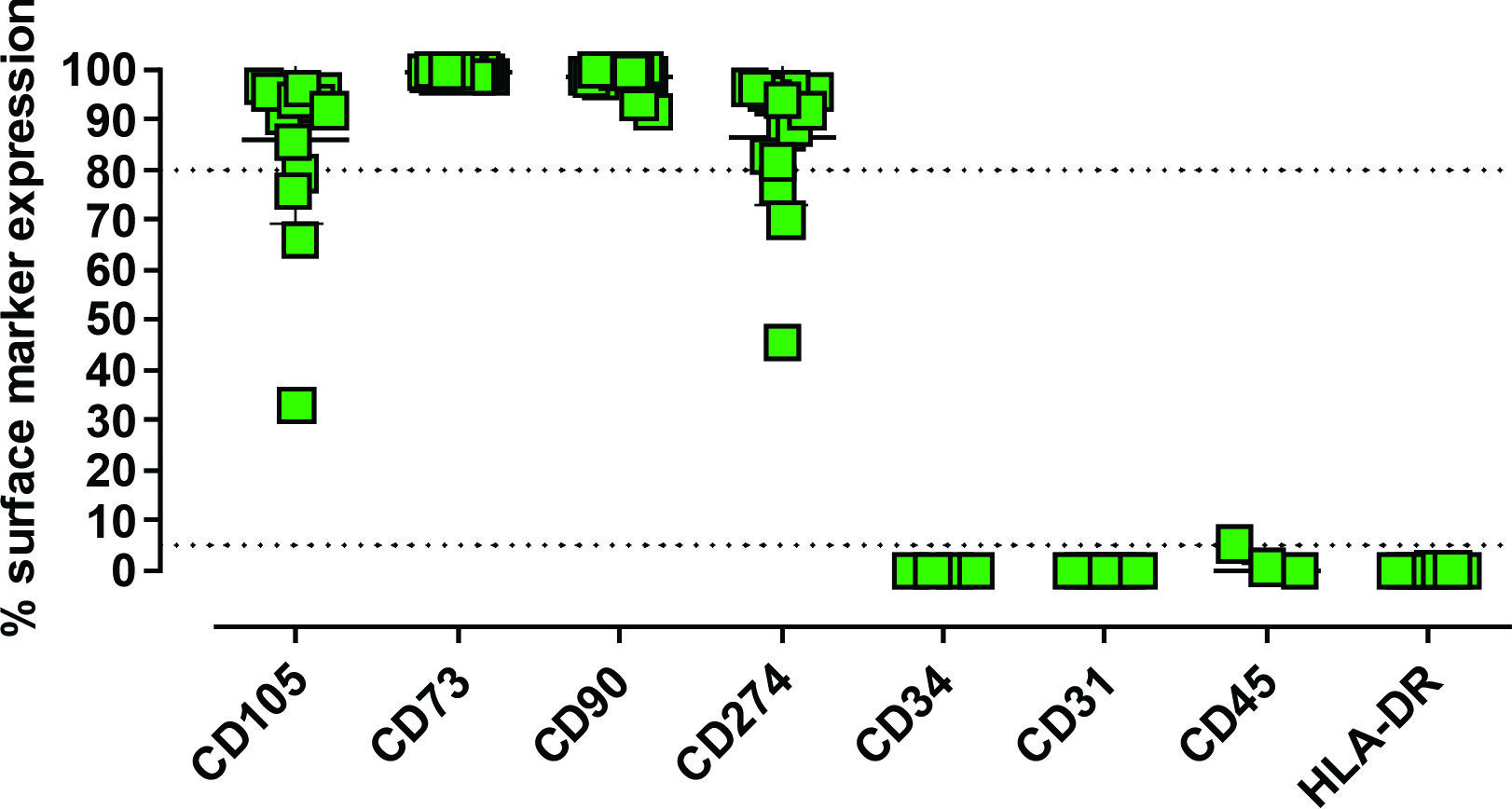

Supplement: Supplementary file 1 [file ijms-21-06284-s001.zip › Figure S1.jpg]

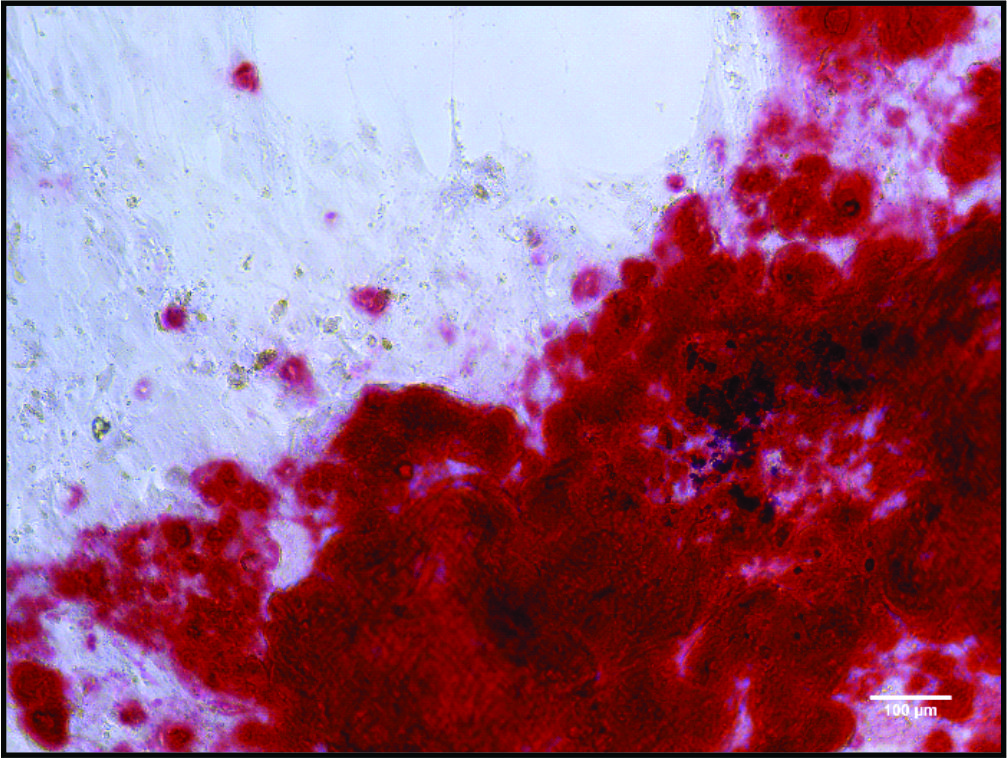

Supplement: Supplementary file 1 [file ijms-21-06284-s001.zip › Figure S2 a.jpg]

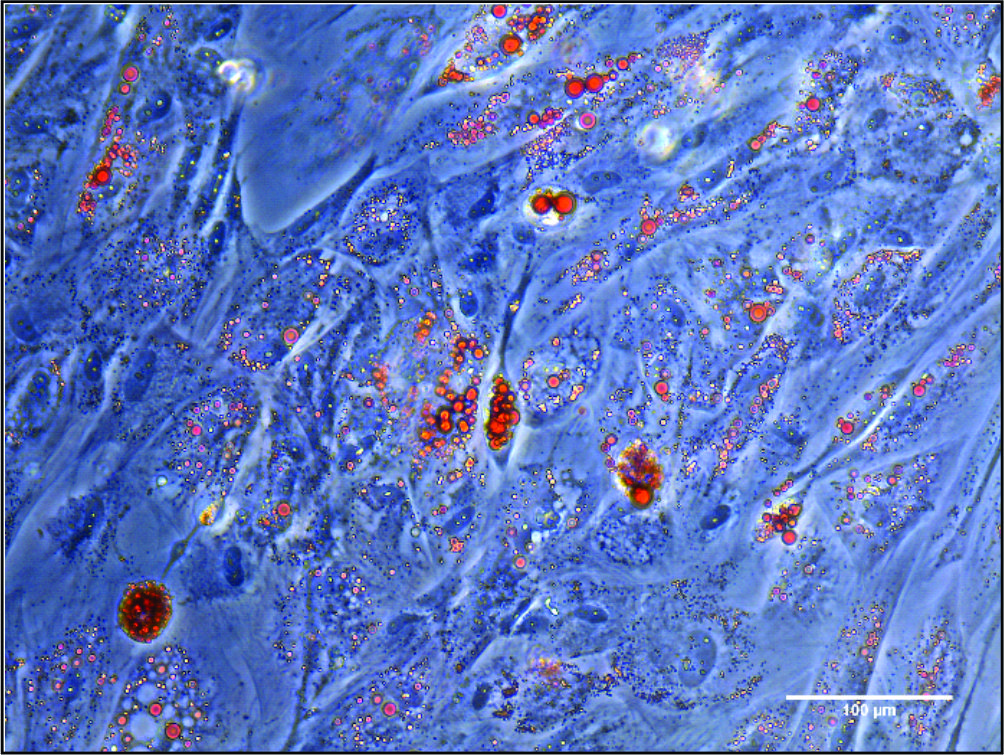

Supplement: Supplementary file 1 [file ijms-21-06284-s001.zip › Figure S2 b.jpg]

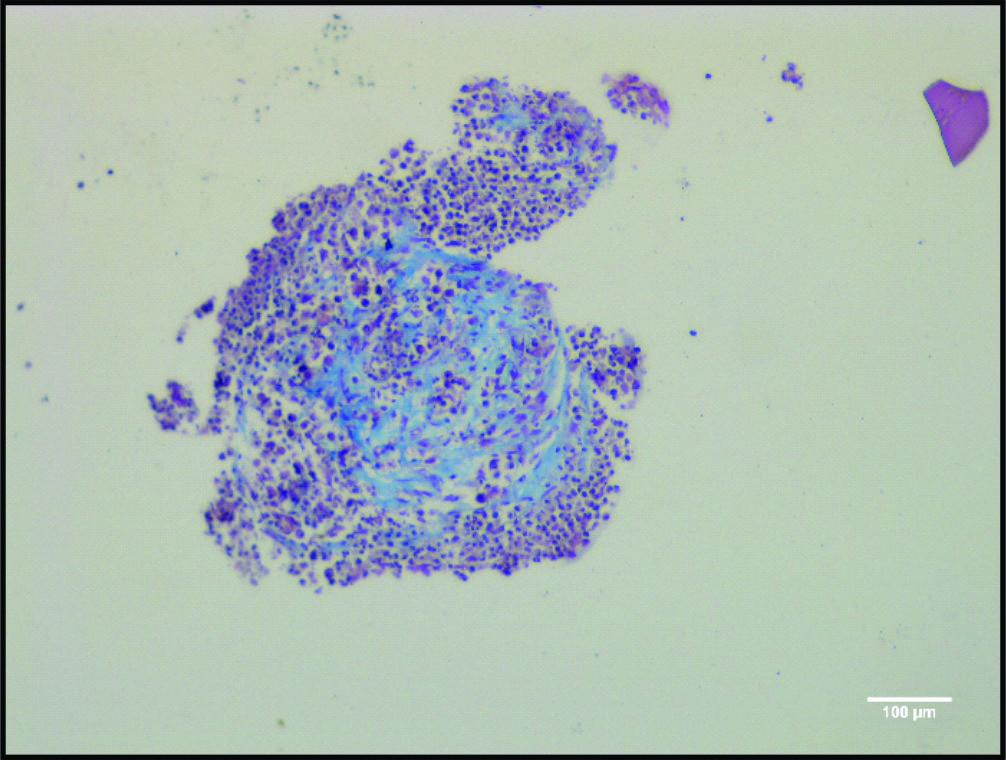

Supplement: Supplementary file 1 [file ijms-21-06284-s001.zip › Figure S2 c.jpg]

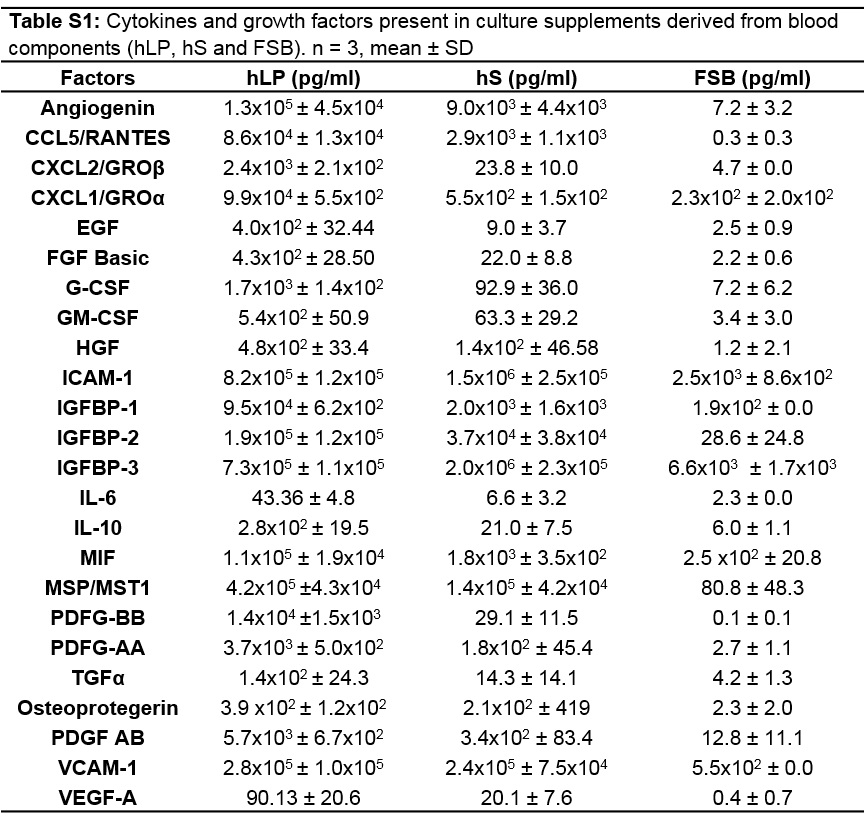

Supplement: Supplementary file 1 [file ijms-21-06284-s001.zip › Table S1.jpg]

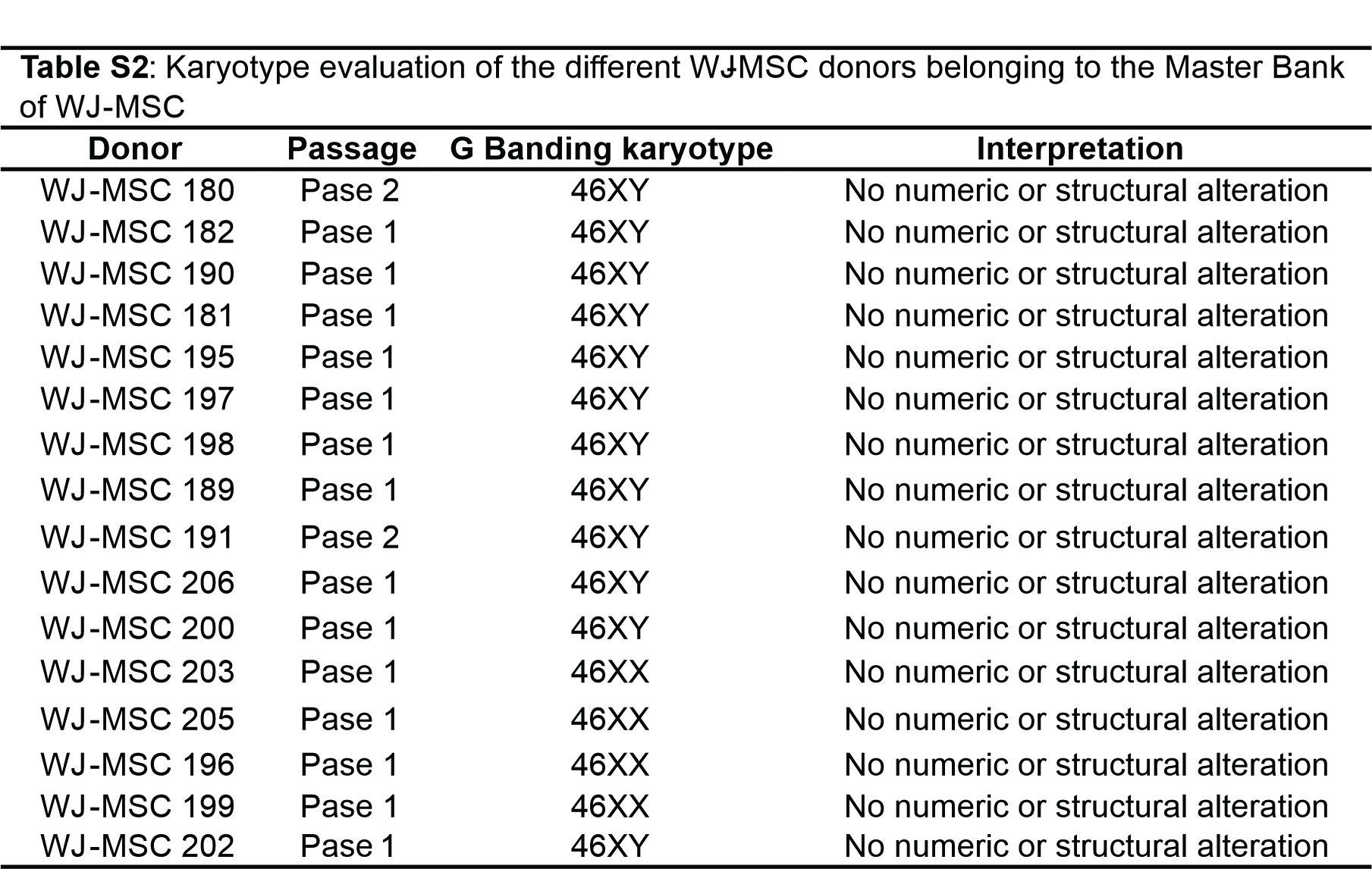

Supplement: Supplementary file 1 [file ijms-21-06284-s001.zip › Table S2.jpg]
